# Supplementary material for: Antiferromagnetic magnonic charge current generation via ultrafast optical excitation
Source: Nat Commun. 2024 May 20;15:4270. doi: 10.1038/s41467-024-48391-1 (PMC11106255; doi:10.1038/s41467-024-48391-1)
Supplement: Supplementary file 1 — Supplementary Information [file 41467_2024_48391_MOESM1_ESM.pdf]

Supporting Information

Antiferromagnetic magnonic charge pumping via ultrafast optical excitation

Lin Huang[1†], Liyang Liao[1,2†], Hongsong Qiu[3†], Xianzhe Chen[4], Hua Bai[1], Lei Han[1], Yongjian Zhou[1], Yichen Su[1], Zhiyuan Zhou[1], Feng Pan[1], Biaobing Jin[3*], and Cheng Song[1*]

[1]Key Laboratory of Advanced Materials (MOE), School of Materials Science and Engineering, Tsinghua University, Beijing, China.

[2] Institute for Solid State Physics, University of Tokyo, Kashiwa, Japan.

[3]Research Institute of Superconductor Electronics (RISE), School of Electronic Science and Engineering, Nanjing University, Nanjing, China.

[4] Department of Materials Science and Engineering, University of California, Berkeley, CA 94720, USA.

Note 1. Néel spin-orbit torques and magnonic charge current generation

It is well established that charge current-induced local spin polarization can couple to the local magnetic moment to produce Néel spin-orbit torque (NSOT), which enables a current-induced rotation of the local moments while preserving their Néel vector [1]. Accordingly, in the reciprocal process of NSOT, a charge current is expected to occur in AFM with local inversion symmetry breaking when staggered non-equilibrium spin polarization is excited by antiferromagnetic magnetization dynamic, i.e., a fluctuation of the Néel vector **n**.

To identify the THz emission mechanisms in Mn_2Au thin films, we measured the time domain THz signals for different linear pump polarization angle α, as shown in Fig. S1. The THz emission signal exhibits independence of the linear pump polarization angle α (**Fig. S1a**), where the azimuth θ of the sample remains the same (see Supporting

Information S2 for THz emission measurement setup). Accordingly, **Fig. S1b** shows an isotropic behavior of the THz signal amplitude on the linear pump polarization (angle α), in contrast to a recent report in (111) NiO thin films [2]. This result suggests that the ultrafast spin-current generation is driving by isotropic excitations in the metallic Mn_2Au being irradiated by the laser pump pulse [3].

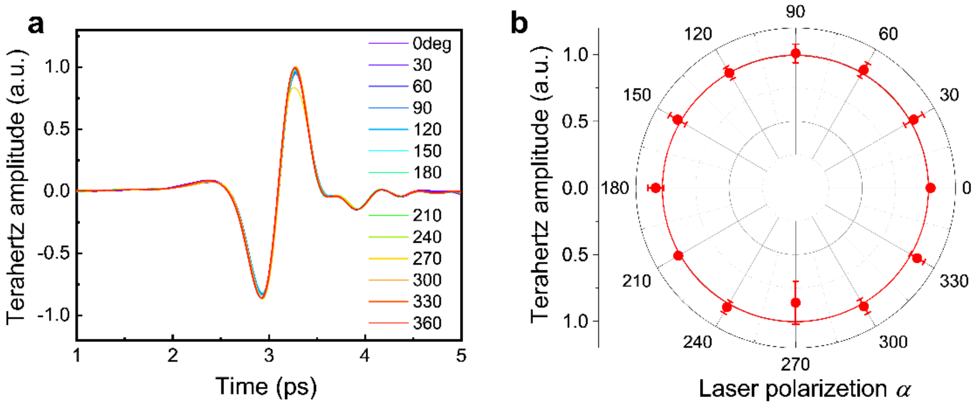

Fig. S1 (a) Time domain THz emission from Mn_2Au with variation of the linear pump polarization angle α. (b) Dependence of the THz signal on the linear pump polarization (angle α) showing an isotropic behavior. Error bars correspond to standard deviations.

In the crystalline structure of Mn_2Au (**Fig. S2a**), the magnetic sublattices exhibit a locally broken inversion symmetry, and the optical pump pulse irradiates the sample surface. The fluctuation of the Néel vector $\boldsymbol{n}$ is excited by the ultrafast optical pulse, resulting in locally non-equilibrium spin polarization with opposite signs on the sublattices. From the initial state illustrated in **Fig. S2b**, Néel vector $\mathbf{n}_0$ is in the initial state before the optical pump irradiation, which is represented in purple. When the optical pump is applied, the Néel vector $\mathbf{n}$(t) experience magnitude reduction due to the optical quenching of the magnetization [4, 5], and the orientation fluctuation in the many-body relaxation process [6] (**Fig. S2c**).

The Holstein-Primakoff transformation for antiferromagnetic magnons indicates spins on site i as $S_{A,i}^{n} = S_0 - a_i^{+} a_i, S_{B,i}^{n} = -S_0 + b_i^{+} b_i$. Here, n labels the equilibrium

Néel vector direction, A and B label the sublattices, a_i, a_i^+, b_i, b_i^+ are the annihilation and creation operators for site i at A and B sublattices, respectively, S_0 is the spin of the moment [7]. When magnon is excited, the spins on A and B sublattices derive from $\pm S_0$, result in the local spin polarization ($\boldsymbol{\sigma}_A = -\boldsymbol{\sigma}_B$), which is equal to the deviations $\sigma\boldsymbol{n}$ of the Néel vector from the equilibrium $\mathbf{n}_0$. Thus, a charge current can be described as $\mathbf{J}_c \sim \boldsymbol{\sigma}_{A,B} \times \hat{\boldsymbol{z}}$ by the reciprocal effect of NSOT, and leads to THz radiation.

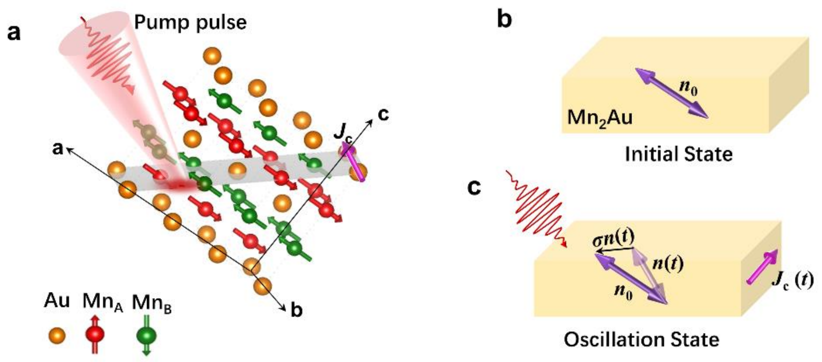

Fig. S2 Generation of magnonic charge current. (a) The crystalline structure of Mn_2Au. Magnetic sublattices Mn_A and Mn_B are marked in red and green, respectively. The projection of magnetic sublattices Mn_A and Mn_B on the Mn_2Au (103) plane aligns along the $[\bar{3}31]$ direction, and laser irradiates on the sample surface. (b)The initial state of Néel vector $\boldsymbol{n}_0$ is represented in purple. (c) Via laser irradiation, a deflection of the Néel vector happens followed by oscillations, i.e. magnon excitations. A magnonic charge current is generated on the Mn_2Au surface by the reciprocal of NSOT, and leads to THz radiation.

Note 2. Schematic of THz emission measurement setup

The schematic of the terahertz emission setup is shown in **Fig. S3**. The linear pump laser polarization angle α is defined with respect to y for the thin film. The pump laser

propagates along the z-axis and sample is set in the x-y plane and the azimuth is denoted by θ. The polarization of the laser-induced THz wave rotates with the rotation of the sample, which is indicated by the angle γ. The y-component of the THz electric field is measured via electro-optical sampling by combining a wire grid polarizer and a (110)-cut ZnTe crystal.

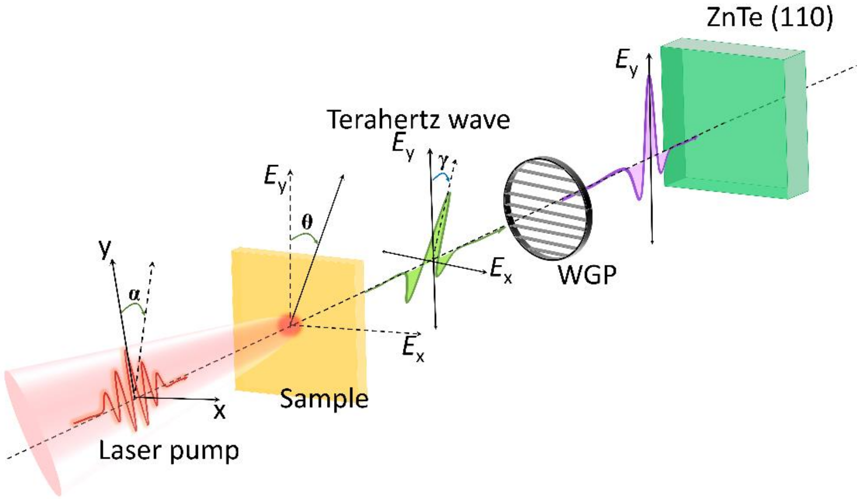

Fig. S3 Schematic of terahertz emission setup, WGP: wire-grid polarizer.

Note 3. Effect of heavy metals on terahertz emission

We utilized two sample preparation methods to create control samples. One method to prevent contamination of the interface is to prepare three samples in situ: Mn_2Au (10 nm), Mn_2Au (10 nm)/Pt (5 nm), and Mn_2Au (10 nm)/W (5 nm). This allows for the transmission of spin currents from the Mn_2Au layer to the heavy metal layer (Method 1). The control THz emission experiments are performed in **Fig. S4a**, Mn_2Au single layer shows the strongest amplitude of THz signal and the polarity of THz signals does not reverse with capping layer W and Pt. Method 2 involves preparing a single layer of Mn_2Au (10 nm) on a relatively large substrate (5 × 10 mm^2). After THz emission measurement from the Mn_2Au single layer, the sample was divided into two identical

pieces (5×5 mm^2) and coated with Pt (5 nm) and metal W (5 nm) respectively, which ensure that the comparison samples are of the comparable antiferromagnetic multi-domain state. The amplitude of Terahertz signal from Mn_2Au single layer, Mn_2Au/Pt and Mn_2Au/W is compared in **Fig. S4b**. The heavy metals Pt and W have opposite spin Hall angles. However, the polarity of terahertz emission does not reverse, and this phenomenon is very similar to the results of previous experiments with the Method 1.

They yield similar results, indicating that capping the heavy metal layer does not improve the THz signal of the Mn_2Au single layer. These results suggest that the strong THz signal emitted by the Mn_2Au single layer is due to the spin charge conversion within the film itself. Whether the spin current is transmitted through the interface layer to enhance the THz signal does not impact our understanding of the high strength of the THz signal emitted by the Mn_2Au single layer.

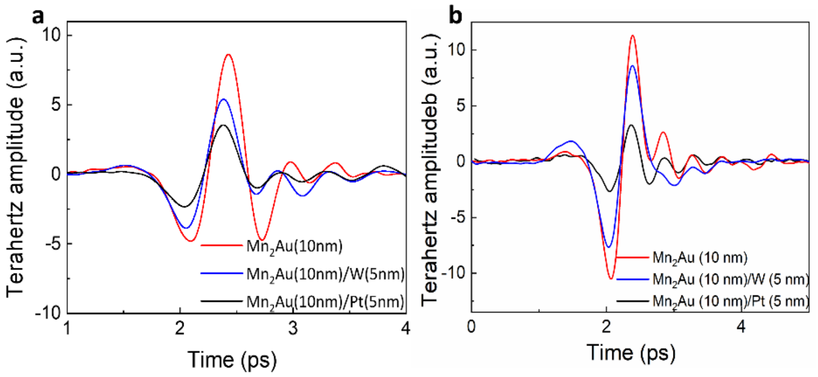

Fig. S4 Terahertz time-domain spectrum of Mn_2Au (10 nm), Mn_2Au (10 nm)/Pt (5 nm) and Mn_2Au (10 nm)/W (5 nm) with Method 1 (a) and Method 2 (b).

Note 4. Thickness dependence of THz emission

The intensity of the terahertz signal emitted by the sample is determined by a random arrangement and distribution of the antiferromagnetic domains of the film, regardless of the thickness. Therefore, we prepared samples with different thicknesses

(11 nm and 15 nm), where four samples for the growth of the 11 nm-thick samples were prepared simultaneously, also two samples for the 15 nm were prepared at the same time. Through terahertz emission experiments, it shows significant differences in the intensity and polarity of terahertz emission in the original state for the four 11 nm samples (**Fig. S5a**). Such a difference was also found in two 15 nm samples (**Fig. S5b**). However, after applying an electric field (+ 4 kV cm^{-1}) to the samples, the terahertz signal intensity of all the samples prepared simultaneously keeps almost the same (11 nm in **Fig. S6a** and 15 nm in **Fig. S6b**). It indicates that the generation of polarized spin is independent of the uncompensated magnetic moment due to the odd set of layers in the original state.

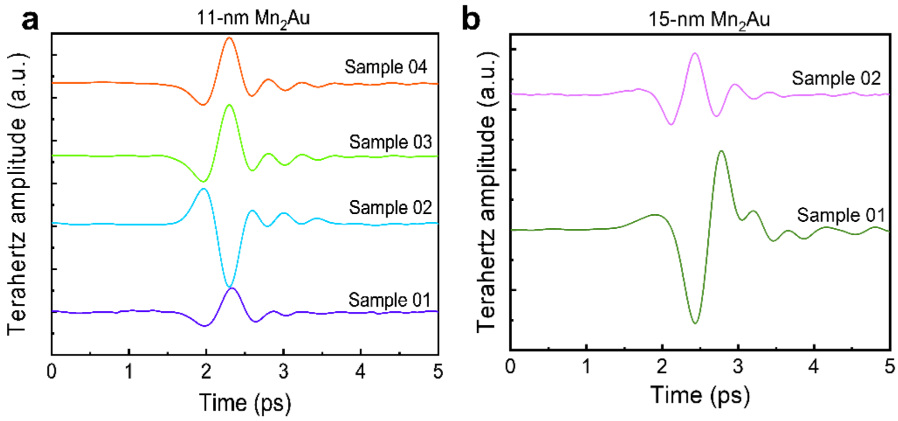

Fig. S5 (a) Terahertz emission for four 11 nm samples. (b) Terahertz emission for two 15 nm samples.

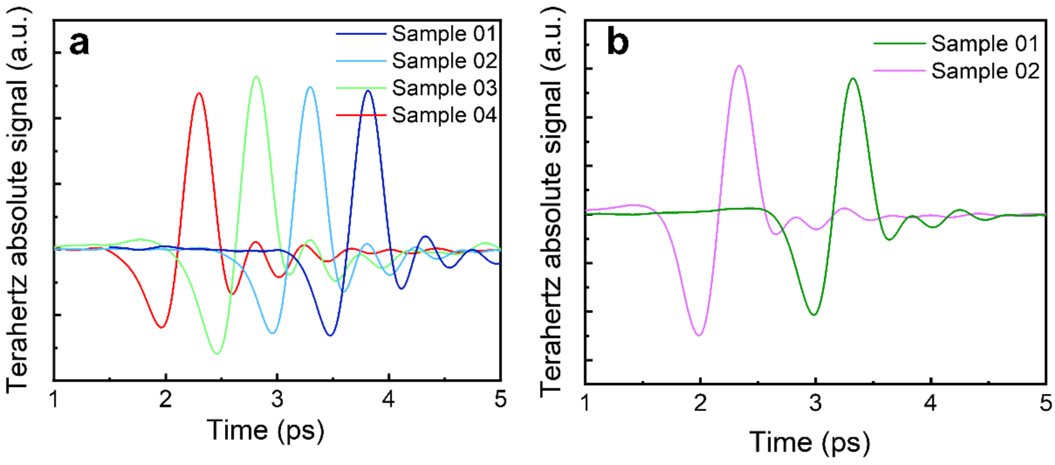

Fig. S6 After applying +4 kV cm^{-1} to the wafers, terahertz absolute signal of (a) four 11 nm samples and (b) two 15 nm samples.

Note 5. Sample growth and characterization

Fig. S7 shows the x-ray diffraction (XRD) spectrum of 15 nm-thick Mn_2Au film grown on PMN-PT (011) substrate. Besides of the diffraction peaks from PMN-PT (011) substrate, only Mn_2Au (103) peak is visible, indicating the growth of (103)-oriented Mn_2Au films. We measured the film samples (100 nm) with XRD ϕ-scans of off-specular Mn_2Au peaks in **Fig. S8a**. The top panel of **Fig. S8a** shows an XRD ϕ-scan of off-specular of Mn_2Au (103) and equivalent peaks of Mn_2Au (013) and down panel of **Fig. S8a** shows an XRD ϕ-scan of off-specular of PMN-PT (011) and equivalent peaks of PMN-PT (111). It indicates two equivalent growth modes of this sample, reflecting the antiferromagnetic multi-domain state for the as-grown sample. This diffraction peak indicates that the material grown by magnetron sputtering is a pure and oriented Mn_2Au phase. The typical high-resolution transmission electron microscopy image of PMN-PT/Mn_2Au (10 nm) is presented in **Fig. S8b**. The white arrow denotes the PMN-PT/Mn_2Au interfaces. It indicates that Mn_2Au is not an epitaxial single crystal sample and the grain size is around 8 nm. However, the lattice structure of Mn_2Au phase can be clearly seen. In the (103) plane of the Mn_2Au films, the $[\bar{3}31]$ axis of Mn_2Au is parallel to [100] axis of PMN-PT (011), as illustrated by the lattice structure in **Fig. S8c** and **Fig. S8d**. In this case, it is reasonable to claim that our sample is a Mn_2Au-(103) textured sample.

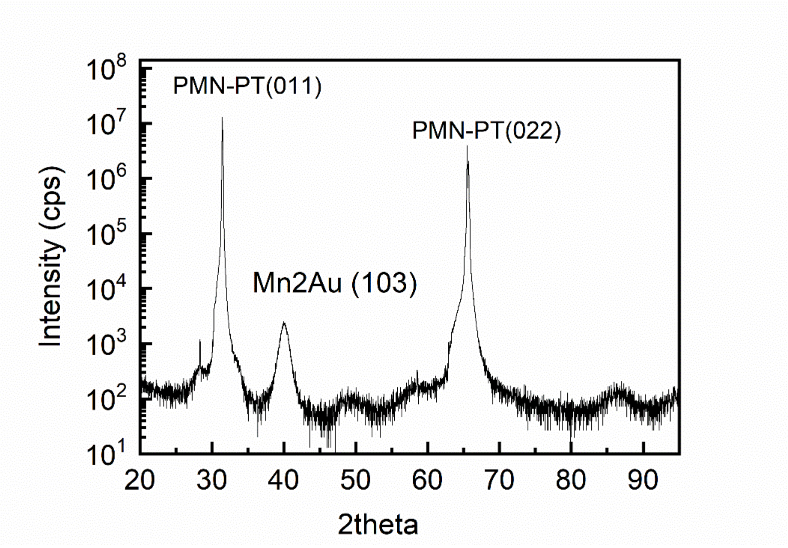

Fig. S7 X-ray diffraction spectrum of Mn_2Au/PMN-PT (011) samples.

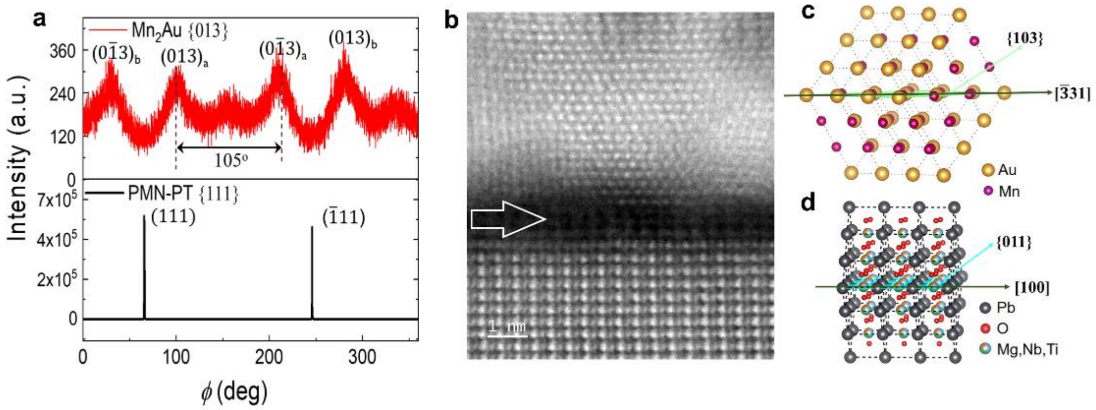

Fig. S8 (a) XRD ϕ-scans of off-specular Mn_2Au-(013), 100 nm thin film (top panel). XRD ϕ-scan of off-specular PMN-PT (111) substrate (bottom panel). (b) z-contrast scanning transmission electron microscopy image of a typical PMN-PT (011)/Mn_2Au (103) cross-section. The scale bar is 1 nm in length. The schematic lattice structures of Mn_2Au (c) and PMN-PT (d) are used to illustrate the crystalline orientation. The (103) plane of Mn_2Au and (011) plane of PMN-PT are highlighted by the green and cyan, respectively.

Note 6. Magnetic properties of Mn_2Au films

Superconducting quantum interference device (SQUID) magnetometry is used to

measure the magnetic properties of the Mn_2Au films on the PMN-PT (011) substrate. The representative magnetization curve of the Mn_2Au (103) film at 300 K is displayed in **Fig. S9**. We can observe typical diamagnetic signals up to 50 kOe, which reflects the diamagnetic feature of the PMN-PT substrate. Hence, the Mn_2Au is an antiferromagnetic film without any ferromagnetic signal.

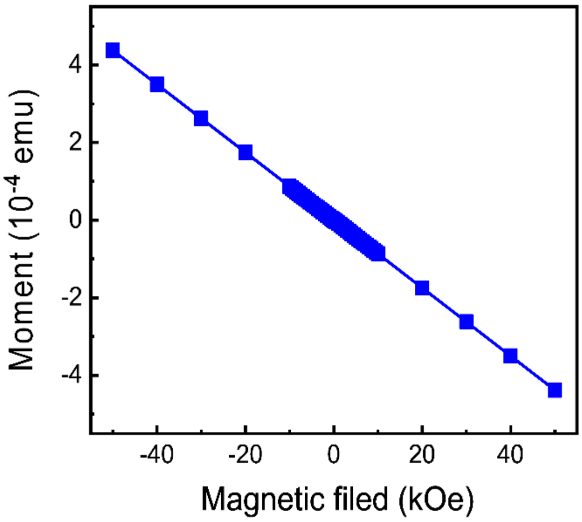

Fig. S9 Room temperature magnetization curve of the Mn_2Au (103) film deposited on the PMN-PT (011) substrate.

References

[1] J. Železný, H. Gao, K. Výborný, J. Zemen, J. Mašek, Aurélien Manchon, J. Wunderlich, Jairo Sinova, and T. Jungwirth, *Relativistic Néel-order fields induced by electrical current in antiferromagnets.* Phys, Rev. Lett. **113**, 157201 (2014).

[2] H. Qiu, L. Zhou, C. Zhang, J. Wu, Y. Tian, S. Cheng, S. Mi, H. Zhao, Q. Zhang, D. Wu, B. Jin, J. Chen, and P. Wu, *Ultrafast spin current generated from an antiferromagnet.* Nat. Phys. **17**, 388–394 (2021).

[3] E. Rongione, O. Gueckstock, M. Mattern, O. Gomonay, H. Meer, C. Schmitt, R. Ramos, T. Kikkawa, M. Mičica, E. Saitoh, J. Sinova, H. Jaffrès, J. Mangeney, S. T. B.

Goennenwein, S. Geprägs, T. Kampfrath, M. Kläui, M. Bargheer, T. S. Seifert, S. Dhillon, and R. Lebrun, *Emission of coherent THz magnons in an antiferromagnetic insulator triggered by ultrafast spin–phonon interactions.* Nat. Commun. **14**, 1818 (2023).

[4] F. Freimuth, S. Blügel, and Y. Mokrousov, *Charge pumping driven by the laser induced dynamics of the exchange splitting*. Phys. Rev. B **95**, 094434 (2017).

[5] K. Krieger, J. K. Dewhurst, P. Elliott, S. Sharma, and E. K. U. Gross, *Laser-Induced Demagnetization at Ultrashort Time Scales: Predictions of TDDFT*. J. Chem. Theory Comput. **11**, 4870 (2015).

[6] W. Töws and G. M. Pastor, *Many-Body Theory of Ultrafast Demagnetization and Angular Momentum Transfer in Ferromagnetic Transition Metals*. Phys. Rev. Lett. **115**, 217204 (2015).

[7] L. Y. Liao, Z. Y. Zhou, Y. J. Zhou, W. X. Zhu, F. Pan, and C. Song, *Charge-magnon conversion at the topological insulator/antiferromagnetic insulator interface.* Phys. Rev. B **102**, 115152 (2020).
